# Ten years trajectories of estimated glomerular filtration rate (eGFR) in a multiethnic cohort of people with type 1 diabetes and preserved renal function

**DOI:** 10.1136/bmjopen-2023-083186

**Published:** 2024-09-10

**Authors:** Salma Ayis, Anastasios Mangelis, Nikolaos Fountoulakis, Julian Collins, Thamer S Alobaid, Luigi Gnudi, David Hopkins, Prashanth Vas, Stephen Thomas, Aicha Goubar, Janaka Karalliedde

**Affiliations:** 1 Population Health Sciences, King's College London, London, UK; 2 King’s Health Partners and School of Cardiovascular Medicine & Sciences, King’s College London, London, UK; 3 King's College Hospital NHS Trust, King's College London, London, UK; 4 King’s Health Partners and King's College London British Heart Foundation Centre of Excellence, School of Cardiovascular & Metabolic Medicine and Sciences, King's College London, London, UK; 5 King's College Hospital NHS Foundation Trust / King's Health Partners, King's College London, London, UK; 6 Diabetes and Endocrinology, King's College Hospital NHS Foundation Trust, London, UK; 7 Guy's and St Thomas' NHS Trust, King’s Health Partners, London, UK

**Keywords:** DIABETES & ENDOCRINOLOGY, EDUCATION & TRAINING (see Medical Education & Training), EPIDEMIOLOGIC STUDIES, STATISTICS & RESEARCH METHODS

## Abstract

**Abstract:**

**Objectives:**

We aim to evaluate estimated glomerular filtration rate (eGFR) patterns of progression in a multiethnic cohort of people with type I diabetes mellitus and with baseline eGFR ≥45 mL/min/1.73 m^2^.

**Design:**

Observational cohort.

**Setting:**

People with a clinical diagnosis of type 1 diabetes, attending two university hospital-based outpatient diabetes clinics, in South London between 2004 and 2018.

**Participants:**

We studied 1495 participants (52% females, 81% white, 12% African-Caribbean and 7% others).

**Primary and secondary outcome measures:**

Clinical measures including weight and height, systolic blood pressure, diastolic blood pressure and laboratory results (such as serum creatinine, urine albumin to creatinine ratio (ACR), HbA1c were collected from electronic health records (EHRs) and eGFR was estimated by the Chronic Kidney Disease–Epidemiology Collaboration. Ethnicity was self-reported.

**Results:**

Five predominantly linear patterns/groups of eGFR trajectories were identified. Group I (8.5%) had a fast eGFR decline (>3 mL/min/1.73 m^2^ year). Group II (23%) stable eGFR, group III (29.8%), groups IV (26.3%) and V (12.4%) have preserved eGFR with no significant fall. Group I had the highest proportion (27.6%) of African-Caribbeans. Significant differences between group I and the other groups were observed in age, gender, HbA1C, systolic and diastolic blood pressure, body mass index, cholesterol and urine ACR, p<0.05 for all. At 10 years of follow-up, 33% of group I had eGFR <30 and 16.5%<15 (mL/min/1.73 m^2^).

**Conclusions:**

Distinct trajectories of eGFR were observed in people with type 1 diabetes. The group with the highest risk of eGFR decline had a greater proportion of African-Caribbeans compared with others and has higher prevalence of traditional modifiable risk factors for kidney disease.

**STRENGTHS AND LIMITATIONS OF THIS STUDY**

- An ethnically diverse cohort of people with type 1 diabetes living in an urban environment provides a large representative sample of a diverse population.
- The use of standardised estimated glomerular filtration rate measures for more than 10 years forms a real-world clinic-based measure with standardised processes.
- Risk stratification of participants using group-based trajectory modelling enhances our understanding of type 1 diabetes-related kidney disease progression in different subgroups which can help inform tailored care and optimisation of resources to address modifiable risk factors before there is a significant loss of kidney function.
- As the study cohort was urban based, it may not represent national cohorts or registry data sets.
- Our data set lacks information on medications and smoking status which is a limitation that may have an impact on results.

## Background

 Type 1 diabetes which affects nearly 10% of all people with diabetes has a significantly higher risk of developing long-term vascular complications as compared with other forms of diabetes. Despite significant advances in diabetes care, people with type I diabetes mellitus (TIDM) can have diminished quality of life and life expectancy often related to the development of vascular complications such as kidney disease. Recent data suggest that despite advances in care a significant proportion of people nearly 40% with type 1 diabetes develop kidney disease over their lifetime.^1 2^

The patterns of progression of estimated glomerular filtration rate (eGFR) decline/change in TIDM have been described in white populations and noted to be heterogeneous with varying rates of progression to advanced stages of chronic kidney disease (CKD).^3 4^ It is increasingly apparent there is a subpopulation of people vulnerable and susceptible to rapid fall in eGFR and faster progression towards end-stage kidney disease (ESKD).^5^

Studies in type 2 diabetes have reported associations between distinct trajectories of eGFR with ethnicity,^6^,^9^ however, no similar data are available in non-white cohorts of people with TIDM. It is also evident that in many people with diabetes, no clinically significant decline in eGFR has been observed over time. In people of white origin with TIDM and proteinuria ‘fast declining’ patterns of eGFR have been described with an interval of 2 to less than 10 years separating normal kidney function to ESKD.^5^ There are no similar studies in ethnically diverse cohorts of people with TIDM.

Our primary hypothesis is that there are distinct trajectories of eGFR in an ethnically diverse population with type 1 diabetes. We retrospectively analysed data from an ethnically diverse cohort of people with type 1 diabetes and preserved eGFR at baseline >45 mL/min/1.73 m^2^ to test our hypothesis. We also assessed differences in the progression of eGFR over time and examined relationships between different patterns of progression and traditional risk factors for kidney disease.

## Methods

The detailed methods and the population studied have been described previously.^10^ In our previous study, we evaluated the baseline risk factors that were associated with a primary endpoint of eGFR fall ≥50% from baseline with a final eGFR <30 mL/min/1.73 m^2^ in a cohort of more than 4000 people with type 1 diabetes. African-Caribbean ethnicity was found to be associated independently of traditional risk factors such as age, duration of diabetes, systolic blood pressure (SBP), retinopathy and albuminuria and independent of socioeconomic status with the primary endpoint of advanced kidney disease progression. In contrast in this manuscript, we report the results of analyses based on our primary hypothesis that there are distinct patterns of eGFR in an ethnically diverse population with type 1 diabetes.

In brief, we included people with a clinical diagnosis of type 1 diabetes as recorded in their primary care, secondary care and diabetes eye screening records, attending hospital clinics in London between 2004 and 2018 who had at least two eGFR measures per year for more than 10 years. Exclusion criteria were pregnancy, eGFR <45 mL/min/1.73 m^2^ at baseline or documented history of non-diabetic kidney disease and age ˂18 years old. Clinical and laboratory data between 2004 and 2018 were collected from electronic health records (EHRs) and included demographics such as self-reported ethnicity, as well as clinical measures such as weight and height, SBP, diastolic blood pressure and laboratory results (such as serum creatinine, urine albumin to creatinine ratio (ACR), HbA1c (glycated haemoglobin, a blood glucose measure) and eGFR estimated by the CKD–Epidemiology Collaboration were all done as per standardised clinical processes in outpatient clinic and a central hospital-based laboratory service.

To create our baseline dataset, we considered the date of the first serum creatinine measurement for each patient as the initial date of entry into the study and extracted all other baseline values within a 2-year span. All other variables that had not been measured within that span were considered missing.

Urinary albumin/ACR was captured from the routine clinical laboratory data. Albuminuria status at baseline was defined as normoalbuminuria, microalbuminuria or macroalbuminuria according to ACR falling in the intervals 0–2.99 (A1), 3–29.99 (A2) or >30 mg/mmol (A3), respectively. Serum creatinine values that were measured during acute hospital admissions were excluded. Serum creatinine was measured at two central laboratories run by the same laboratory services provider using an isotope dilution mass spectrometry-traceable modified enzymatic method on the Roche 702 platform (Roche, Basel, Switzerland). ACR was measured in urine samples taken from routine clinical care using immunoturbidimetry for albumin and enzymatic method for creatinine Roche 702 platform (Roche, Basel, Switzerland). This retrospective study was conducted in line with local audit protocols using existing anonymised routine clinical data that was only accessed directly by the clinical care teams and the project was approved by Guys and St. Thomas hospital and Kings College Hospital data governance committees.^10^

### Patient and public involvement

As data for this study were based on hospitals electronic health records (EHRs), patients were not directly involved in the research design. Outcome measures were all done as per standardised clinical processes in outpatient clinic and a central hospital-based laboratory service. The results will be publicly disseminated to conferences, seminars and peer-reviewed journals.

### Statistical analysis

Data of the full cohort were summarised using mean and SD, or medians and IQR for continuous variables, frequencies and proportions for categorical variables. Group-based trajectory modelling (GBTM), a special class of finite mixture modelling, was used to identify developmental trajectories of eGFR over 10 years.^11^,^13^ The method uses formal statistical tests to identify (rather than assume a priori) distinctive trajectories for subgroups that follow similar developmental course of the outcome over time. The procedure starts with simple assumptions about the number of groups and the shape of eGFR progression for each, then sequentially examines a range of number of groups and various shapes of progression parameters for each, until an optimal solution is reached. The optimal solution for this study has been determined by recommended criteria that included formal statistical tests such as the Bayesian information criterion (BIC), Akaike information criteria (AIC); the model with the lowest value of BIC and AIC, is preferred.^14^ The final choice between competing models was further moderated by an entropy (a statistical test that indicates the accuracy of the classification) approaching 1.0 and greater than 0.9^15^; average posterior probability value >0.90 for group membership; the parsimony of model that fits data well, with all parameters being significant, and the sample size within each group being no less than 5.0%. A wide range of models was tested with different shapes of progression parameters, including constant, linear and quadratic.

The method is based on probabilities, and it assigns a probability of group membership to each individual using a maximum likelihood approach.^16^ GBTM was undertaken using a Stata Plugin algorithm, eGFR was modelled using the censored normal distribution.^17^

We excluded participants who were missing eGFR at baseline, at the final follow-up point, and those with less than two observations of eGFR over 10 years. The GBTM methods handle missing data under the assumption that data are missing at random.^18^

The characteristics of the final identified groups at baseline were compared initially using one-way analysis of variance for continuous variables that met the normality distribution assumption and Kruskal-Wallis for continuous variables that did not (ACR). A χ^2^ test was used for categorical variables. In addition, we used linear regression to compare the trajectory groups adjusting for age, gender and ethnicity. The goodness of fit of the regression models was assessed by the normality of the residuals’ distribution. Sensitivity analyses were performed first, on data with none missing (complete case analysis, CCA), one, two, three or four observations missing using kappa and weighted kappa, to assess agreement between final models and models based on less missing data by means of internal validation. Second, individual slopes were derived using linear regression, excluding observations with eGFR ≤15 mL/min/1.73 m^2^ somewhere in the middle of the follow-up, and examining the distribution of the slopes for each of the final groups identified by the GBTM. Shape parameters of eGFR and regression coefficients (slopes) were assessed using the standard Ward test.^14^ The threshold alpha (α=0.05) was used to determine statistical significance. Exploratory profile plots were used to illustrate eGFR decline in patients who reached either stage 4 or stage 5 CKD by 10 years of follow-up from each of the final trajectories aiming to test whether the progression in these subgroups was predominantly linear or non-linear. All analyses were undertaken using Stata (V.17.0) for Windows.^19^

## Results

The baseline features of the cohort are shown in table 1. Mean age was 43.75 years SD of 13.55 years and 53% of the cohorts were female. Ethnic distribution was 81.1% white, 2.5% Asian, 12% African-Carribean and 4.4% others or mixed. Clinical characteristics are summarised in the table 1.

**Table 1:** Baseline clinical and biochemical characteristics of 1495 people with type 1 diabetes

Variable name	Mean (SD)
Age (years)	43.75 (13.55)
Duration of diabetes (years)	16.86 (14.05)
Gender	
Female, n (%)	783 (52.37)
Male, n (%)	712 (47.63)
Ethnicity, n (%)	
White	1212 (81.07)
African-Caribbean	180 (12.04)
Asian	38 (2.54)
Mixed/other	65 (4.35)
Weight (kg)	74.06 (15.6)
BMI (kg/m^2^)	25.74 (4.61)
eGFR (mL/min/1.73 m^2^)	83.22 (21.2)
SBP (mm Hg)	125.13 (16.07)
DBP (mm Hg)	74.2 (9.02)
HbA1c (mmol/mol)	70.86 (22.25)
Cholesterol (mmol/L)	4.75 (1.05)
HDL-cholesterol (mmol/L)	1.63 (0.49)
LDL-cholesterol (mmol/L)	2.57 (0.86)
Triglycerides (mmol/L)	1.28 (0.97)
Urine ACR (mg/mmol)	28.77 (38.45)
Albuminuria status/grade	n (%)
A1 (ACR<3): n, %	282 (18.86)
A2 (ACR 3–30): n, %	670 (44.82)
A3 (ACR>30): n, %	543 (36.32)

Range (lower quartile (25th centile), upper quartile (75% centile)), IQR.

ACRalbumin to creatinine ratioBMIbody mass indexDBPdiastolic blood pressureeGFRestimated glomerular filtration rateHbA1Cglycated haemoglobin, measurement of sugar in the bloodHDLhigh-density lipoproteinLDLlow-density lipoproteinSBPsystolic blood pressure

**Figure 1:**
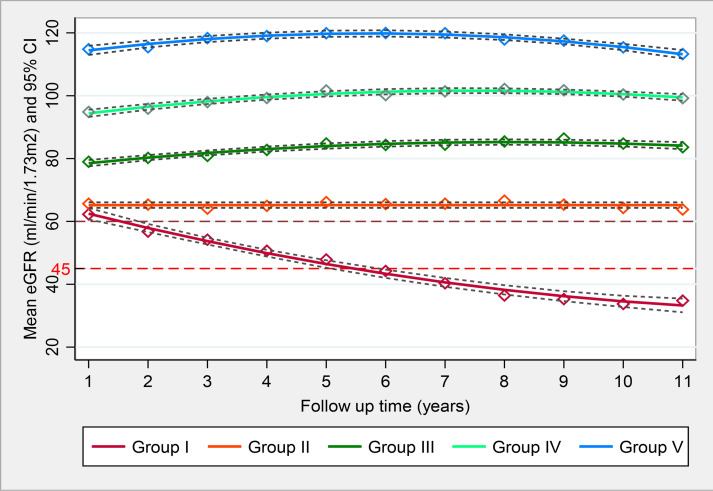
Trajectories of eGFR (Estimated Glomerular Filtration Rate) over 10 years of follow-up in people with type I diabetes (Five different patterns of progression of eGFR mL/min/1.73 m² over 10 years).

The optimal solution of the GBTM analyses provided five qualitatively distinct developmental trajectories of eGFR over 10 years of follow-up. The estimates of the shape parameters of the trajectories are shown in table 2 and figure 1. Group I has a negative linear slope of −5.09 (SE of 0.48 mL/min/1.73 m^2^/year) indicating a rapid decline in eGFR. Group II has low, stable eGFR over time, intercept 65.19 (SE:0.44). Groups III, IV and V had similar shapes, with varying intercepts of 76.51 (0.68), 92.0 (0.77) and 111.92 (1.02), positive linear slopes >2 each and a quadratic parameter of a small negative magnitude, indicating slow/moderate increase, followed by the minimal decline in eGFR suggestive of no clinically meaningful change. The mean posterior probabilities (an index of probability of group membership) for all groups were very high at 0.96 for group I, 0.95 for group II, 0.94 for each of groups III and IV, and 0.97 for group V. The probabilities for membership to other groups were very low indicating very good classification of the groups^20^ (online supplemental table S1).

**Table 2:** Estimated parameters of developmental trajectories of eGFR over 10 years of follow-up in 1495 people with type 1 diabetes

Group	Parameter	Estimate	SE	P value
I	Intercept	67.37	1.30	<0.001
	Linear	−5.09	0.48	<0.001
	Quadratic	0.18	0.04	<0.001
II	Intercept	65.19	0.44	<0.001
III	Intercept	76.51	0.68	<0.001
	Linear	2.17	0.25	<0.001
	Quadratic	−0.13	0.02	<0.001
IV	Intercept	92.00	0.77	<0.001
	Linear	2.59	0.27	<0.001
	Quadratic	−0.17	0.02	<0.001
V	Intercept	111.92	1.02	<0.001
	Linear	2.76	0.37	<0.001
	Quadratic	−0.24	0.03	<0.001

The baseline characteristics for each of the groups are presented in table 3. Group I and group II comprised 8.5% and 23% of the cohort and had lower eGFR than others at baseline. The mean (SD) of eGFR for the five groups (I–V), respectively, were 66.15 (17.49), 68.10 (11.9), 80.74 (13.64), 96.31 (14.43) and 115.87 (16.18) mL/min/1.73 m^2^, respectively.

**Table 3:** Comparison of baseline clinical and biochemical characteristics of 1495 people with type 1 diabetes classified by group membership as identified by a group-based trajectory modelling

Variable name	Group I	Group II	Group III	Group IV	Group V	P value
Group size (%)	127 (8.45)	344 (23.01)	446 (29.83)	393 (26.29)	185 (12.37)	
Age (years)	50.93	50.84	45.44	39.1	31.46	<0.001
(SD)	15.13	13.57	11.97	10.74	7.97	
Duration of diabetes (years)	15.59	17.99	16.87	17.13	14.96	0.152
(SD)	15.85	15.64	14.35	12.96	10.6	
Female %	60	116	194	254	159	<0.001
	47.24	33.72	43.5	64.63	85.95	
Male%	67	228	252	139	26	
	52.76	66.28	56.5	35.37	14.05	
White	87	249	375	344	157	
	68.5	72.38	84.08	87.53	84.86	
African-Caribbean	35	71	46	22	6	
	27.56	20.64	10.31	5.6	3.24	
Asian	3	12	8	8	7	<0.001
	2.36	3.49	1.79	2.04	3.78	
Mixed/other	2	12	17	19	15	
	1.57	3.49	3.81	4.83	8.11	
Weight (Kg)	75.75	77.51	75.44	72.21	67.11	0.001
(SD)	16.43	14.93	15.12	14.36	17.26	
BMI (kg/m^2^)	26.47	26.25	25.71	25.54	24.76	0.002
(SD)	5.28	4.36	4.34	4.63	4.96	
eGFR (mL/min/1.73 m^2^)	62.58	65.44	79.06	95.21	115.02	<0.001
(SD)	15.92	11.03	12.57	13.66	14.78	
SBP (mm Hg)	131.89	128.64	125.99	121.87	118.82	<0.001
(SD)	17.33	16.75	16.17	14.29	13.45	
DBP (mm Hg)	76.06	74.04	74.3	74	73.36	0.118
(SD)	10.4	8.83	8.83	8.9	9.03	
HbA1C (mmol/mol)	78.37	69.07	69.95	69.71	73.63	<0.001
(SD)	27.38	22.43	22.13	20.27	21.27	
ACR (mg/mmol)						<0.001
Median (Range)	39 (11.8; 45))	18.5 (7; 44)	12 (4.1: 42)	9 (3.2; 40)	7.7 (2; 32)	<0.001
Cholesterol (mmol/L)	4.84	4.77	4.78	4.73	4.6	0.265
(SD)	1.28	1.1	1.06	0.98	0.95	
HDL (mmol/L)	1.64	1.6	1.59	1.65	1.71	0.057
(SD)	0.67	0.49	0.45	0.47	0.45	
LDL (mmol/L)	2.59	2.51	2.6	2.62	2.47	0.224
(SD)	1.07	0.86	0.85	0.83	0.81	
Triglyceride (mmol/L)	1.42	1.35	1.27	1.24	1.18	0.004
(SD)	0.81	1.1	1.01	0.95	0.75	
Albuminuria status/grade						
A1 (ACR<3): n	10	40	86	92	54	<0.001
%	7.87	11.63	19.28	23.41	29.19	
A2 (ACR 3–30): n	43	152	203	189	83	<0.001
%	33.86	44.19	45.52	48.09	44.86	
A3 (ACR>30): n	74	152	157	112	48	<0.001
(%)	58.27	44.19	35.20	28.50	25.95	

Range (lower quartile (25th centile), upper quartile (75% centile)).

ACRalbumin to creatinine ratioBMIbody mass indexDBPdiastolic blood pressureeGFRestimated glomerular filtration rateHbA1c glycated haemoglobin, measurement of glucose in the bloodHDLhigh-density lipoproteinLDLlow-density lipoproteinSBPsystolic blood pressure

The five trajectory groups also had differences at baseline in age, ethnicity, gender, HbA1c, SBP, urine ACR and Triglyceride (p≤0.05). Group I had higher SBP, diastolic blood pressure, HbA1C and ACR compared with others (p<0.05 for all), and the group had the highest prevalence of people of African-Carribean ethnicity comprising 27.56%, followed by group II, 20.6% and groups III, IV and V, having 10.31%, 5.6% and 3.2%, respectively. Similarly, the prevalence of white ethnicity was lowest in group I, 68.5%, compared with 72.38%, 84.08%, 87.53% and 84.86% in groups II–V, respectively. The proportion of females was less than 50% in groups I–III, but greater than 50% in groups IV and V, with the latter having 86% females. Significant differences remain after adjusting for age, gender and ethnicity using multivariable linear regression models (online supplemental table S2).

Further analyses performed to explore the robustness of our results that involved the full data including cases with missing observations, in comparison with the CCA and other options of less missingness, demonstrated good agreement with the final five groups’ solution. Agreement between classifications has shown high kappa and weighted kappa (online supplemental tables S3 and S4). The distribution of individual slopes excluding observations that reached eGFR ≤15 mL/min/1.73 m^2^ was also consistent with the slopes reached by the GBTMs (online supplemental table S5 and figure S1).

Longer-term eGFRs decline (10 years) among the different groups, and the chance of reaching advanced CKD stages varied remarkably across the five groups. The proportions of people reaching CKD stage 3B (eGFR 30–45 mL/min/1.73 m^2^), for example, was 71.65% in group I, 5.8% in group II and <1% to none in the other groups. Similarly, 34% of group I and only 1.2% of group II reached stage 4 CKD (eGFR 15–30 mL/min/1.73 m^2^), and only people from group I progressed to stage 5 CKD (eGFR<15 mL/min/1.73 m^2^) with 16.5% reaching the stage (online supplemental table S6).

**Figure 2:**
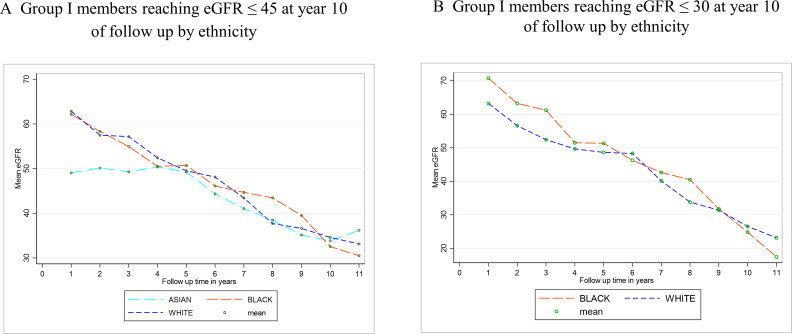
Decline in eGFR by group and by ethnicity. The figure describes group I members decline over 10 years by ethnicity; (**A**) those who declined to reach eGFR≤45 mL/min/1.73 m² and (**B**) eGFR≤30 mL/min/1.73 m² over 10 years. eGFR, estimated glomerular filtration rate.

The profile plots of eGFR for the subgroups who reached stage 4 or stage 5 CKD suggest a linear decline. Patterns of decline are generally similar across all ethnic groups in group I, as expected in consistency with the model parameters (figure 2A,B).

## Discussion

In a multiethnic cohort of people with type 1 diabetes and preserved kidney function, followed for 10 years with serial eGFR measurements, five groups of distinctive patterns of eGFR progression were observed. Most other studies that have evaluated eGFR trajectories have studied white people with type 1 diabetes and albuminuria.^3 21^ In these studies, only selecting people with albuminuria would have an enhanced risk of eGFR decline. In contrast, we studied people with eGFR >45 mL/min/1.73 m^2^ at baseline and thus observed distinct patterns of progression and no progression of eGFR. Our finding that the group with the greatest proportion of African-Caribbean people also had the highest risk of rapid decline in eGFR and consequently the worst long-term renal outcomes are of major clinical importance, with 34% of this group developing stage 4 CKD and 16% stage 5 CKD. Of note, people in this highest-risk group had mean eGFR fall of 5 mL/min/1.73 m^2^ indicative of fast progression of CKD. There were also significant differences between group I and the other groups observed for traditional risk factors for CKD progression such as age, gender, HbA1C, SBP and diastolic blood pressure, body mass index (BMI), cholesterol and albuminuria.^22^ Although the general consensus is that eGFR linearly changes overtime, the non-linear (quadratic) terms indicate a decrease in the eGFR slope as time progresses which is reflected by a modest rise in eGFR over time which is unlikely to be of clinical significance. Further, it is well established that eGFR values >60 mL/min are a less accurate reflection of true renal function when compared with gold-standard measures of GFR measurement and these observed trends in rise in eGFR may reflect the inherence inaccuracies of eGFR equations.

Overall, our data suggest that most of our cohort (groups II, III, IV and V) had reasonably stable eGFR within a normal range with mild fluctuations of modest clinical impact. Similar data have been observed in other studies, although in preselected CKD cohorts where 15.3% of the sample were classified as improvers.^23 24^ Another study where, participants were newly diagnosed with hypertension, whose eGFR was at CKD stages G1–G2, have initiated ACEIs, ARBs, beta-blockers, calcium channel blockers or diuretics (Ds) as first-line antihypertensive monotherapy, has concluded that eGFR was almost maintained at baseline level, in the first follow-up year, compared with dropping 3 mL/min/1.73 m^2^ per year before drug initiation.^25^ While in a study of people with early-stage CKD monitored in primary care, eGFR rose over time in nearly 41.3% of the cohort.^26^

Similarly in 1441 participants with type 1 diabetes, in the Diabetes Control and Complications Trial, 149 people (10.4%) had evidence of early eGFR loss defined as slope ≤ − 3% per year over a mean of 6.5 years.^27 28^ Of note most people in this cohort (86%) were normoalbuminuric and baseline mean eGFR was 117 mL/min/1.73 m^2^, suggesting a lower renal risk cohort as compared with ours. Furthermore, this cohort was predominantly (>99%) of white origin.

Studies in higher-risk cohorts of type 1 diabetes (all participants had macroalbuminuria) also demonstrate heterogeneity in rates of eGFR decline over time. In an observational study of 364 people with type 1 diabetes who reached ESKD, the rate of eGFR decline per year ranged from −72 to −2 mL/min/1.73 m^2^. The majority (nearly 87%) of the trajectories were characterised with linear regression-based spline models.^5^ In a cohort of 161 people with type 1 diabetes and proteinuria, in less than one-third, eGFR declined with widely different slopes, ESKD was developed between 2 and 18 years of follow-up, and early eGFR slopes predicted the risk of ESKD better than traditional risk factors such as HBA1c, urine ACR and blood pressure even when renal function was within normal ranges.^21^

In our cohort, we did not select people who reached ESKD only nor did we select only those with albuminuria. Our results, however, are consistent with the above observations in that the predominant feature of eGFR trajectory in those who declined over time was linear (group I) and that this group had a greater prevalence of known risk factors for progression such as higher HbA1c, urine ACR, raised triglycerides, SBP and diastolic blood pressures. In comparing the two dominant ethnicities of group I (African-Caribbean and white) at baseline in all the known traditional risk factors, no differences were found with exception of diabetes duration where the white have a much longer duration 18.33 (95% CI 12.88 to to 18.69) compared with the African-Caribbeans, whose average duration was 9.77 (95% CI 6.35 to to 13.19) years.

Distinct from the above observations we also noted a greater prevalence of people of African-Caribbean origin in the ‘high-risk’ group I that had an annual fall of eGFR >5 mL/min/1.73 m^2^ year. These results are consistent with our data from the same cohort where we reported the independent impact of African-Caribbean ethnicity in decline in eGFR >50% from baseline with final eGFR <30 mL/min/1.73 m^2^.^10^

Most of the data for kidney disease in type 1 diabetes come from studies in white cohorts, and there is indeed no information on the risks of progression of CKD in people with type 1 diabetes from other ethnicities.^10^ The exact mechanism for our observations of this enhanced risk in people of African-Caribbean ethnicity with type 1 is unclear. There is evidence that people of African-Caribbean heritage with type 2 diabetes or hypertension are predisposed to premature kidney disease but the exact mechanisms and possible causal factors for this susceptibility to kidney complications are poorly understood with conflicting views.^29^

This study was not designed to identify the putative reasons or mechanisms that explain our observations on the impact of ethnicity on the observed eGFR group trajectories. We can speculate, however, on several possible explanations for our results which include the detrimental impact of historical suboptimal glycaemic control. In paediatric cohorts of people with type 1 diabetes, higher HbA1c in African-Caribbean children with type 1 diabetes compared with their white counterparts but, were unable to assess the impact of HBA1c and ethnicity on advanced kidney or other microvascular complications due to their design.^30 31^

People with diabetic kidney disease may have features of insulin resistance and often have a tendency to greater salt sensitivity and its related vascular injury. Whether these pathophysiological features are more pronounced in African-Caribbean people with type 1 diabetes requires further investigation.

To our knowledge, this is the first study to use GBTMs in a multiethnic population of type I diabetes, aiming to explore the heterogeneity in eGFR progression. Unlike conventional mixed effects models that assume all individuals are drawn from a single population and have a single growth curve, GBTMs recognise the heterogeneity in the population and detect various patterns of development over time.^12 32^ Variety of similar approaches that identify latent classes or trajectories have been applied to understand the development of outcomes in patients of different health conditions, including the progression of HbA1c in youth with type 1 diabetes and eGFR in adults with type 2 diabetes.^33 34^ The methods have proven to be helpful in identifying trajectories of disease processes and clustering of associated factors with individual group trajectories.^35^

### Strengths and limitations

The strengths include the study’s contemporaneous nature and having a cohort with ethnic diversity representative of urban-dwelling people with type 1 diabetes. The use of standardised eGFR measures for more than 10 years, real-world clinic-based measures and assessments, laboratory data from a single unit with standardised processes are further strengths of our work.

The clinical implications of our results are that early changes in eGFR trajectories may enable clinicians to identify higher-risk groups more promptly before there is a significant loss of kidney function. Such early prompt risk stratification may enable more enhanced care, resources and optimisation of evidence-based treatments known to slow kidney disease and associated cardiovascular disease in those at highest need and risk in resource and time-stretched healthcare systems.

The limitations of our study are that our cohort is from an urban environment, and therefore, unlikely to be representative of national cohorts or registry data sets. A major limitation of our study is the lack of data and information on medications and smoking status. These data were not captured in our study and we cannot, therefore, exclude that the observed results may be related to differences in the use of medications including medications such as RAS inhibitors that have demonstrated renal benefits.^27 28^ Our study was not designed to evaluate the potential pathophysiological mechanisms that may explain our findings. However, we can speculate that salt sensitivity and insulin resistance are harbingers for progressive kidney decline in type 2 diabetes may be more pronounced in the high-risk group we identified. It is also possible that observed differences may be related to a historical legacy of poor glycaemic control and thus a prolonged high mean glycaemic exposure that can predispose people to progression of kidney disease directly or via epigenetic mechanisms.^27^ We had no formal laboratory confirmation of diabetes subtype and relied on EHRs and self-reporting of diabetes status when people attended for eye screening. It is possible that people with ketosis-prone diabetes may have been labelled with type 1 diabetes. However, in our previous work from this cohort, we did not observe any significant differences in features such as higher BMI, greater body weight or older age, which may be more prevalent in the ketosis-prone phenotype.^10^

In conclusion, in a multiethnic population of 1495 people with type 1 diabetes with preserved renal function at baseline, we observed five distinct patterns of eGFR trajectories. The group with the highest risk of a clinically significant fast eGFR decline (annual eGFR >5 mL/min) had a greater proportion of African-Carribean people compared with other groups as well as higher prevalence of traditional risk factors for kidney disease. Further longer-term studies in similar ethnically heterogeneous cohorts of people with type 1 diabetes are needed to identify potential mechanisms that explain our observations on heterogeneity of eGFR decline and related trajectories.

## supplementary material

10.1136/bmjopen-2023-083186 online supplemental file 1

## Data Availability

Data may be obtained from a third party and are not publicly available. Subject to local data acquision regulations.
