# Supplementary material for: Ten years trajectories of estimated glomerular filtration rate (eGFR) in a multiethnic cohort of people with type 1 diabetes and preserved renal function
Source: BMJ Open. 2024 Sep 10;14(9):e083186. doi: 10.1136/bmjopen-2023-083186 (PMC11409247; doi:10.1136/bmjopen-2023-083186)
Supplement: online supplemental file 1 [file bmjopen-14-9-s001.pdf]

Table of Contents

**Titile**	**Description**	**Page no.**
**Table S1**	Posterio probabilities of group membership	2
Table S2	Adjusted[§] estimates of diffrences of baseline clinical indicators between Group I and other trajectories (Group II, III, IV and V)	3-4
Table S3	Summary of missing data (out of 11 points) over time by trajectory	5
Table S4	Kappa and weighted Kappa to assess the agreement of the classification achieved by the final model and other models using complete data and less number of missing	6
Table S5	Sumarry of individiual slopes (excluding observations that follow eGFR ≤ 15 ml/min/1.73m^2) for each of the five groups	7
Table S6	Proportions of people with Type I diabetes reching various CKD stages over 10 years of follow up by trajectory	8
Figure S1	Historgrams of individual slopes of eGFR over 10 years by trajectories (groups)	9

Table S1. Posterio probabilities of group membership

Group	n	Mean	SD	Min	Max
**I**	**127**	**0.96**	**0.11**	**0.52**	**1.00**
II	127	0.04	0.11	0.00	0.48
III	127	0.00	0.00	0.00	0.00
IV	127	0.00	0.00	0.00	0.00
V	127	0.00	0.00	0.00	0.00
I	344	0.01	0.06	0.00	0.48
**II**	**344**	**0.95**	**0.12**	**0.50**	**1.00**
III	344	0.04	0.10	0.00	0.50
IV	344	0.00	0.00	0.00	0.00
V	344	0.00	0.00	0.00	0.00
I	446	0.00	0.00	0.00	0.00
II	446	0.03	0.08	0.00	0.49
**III**	**446**	**0.94**	**0.11**	**0.50**	**1.00**
IV	446	0.04	0.09	0.00	0.50
V	446	0.00	0.00	0.00	0.00
I	393	0.00	0.00	0.00	0.00
II	393	0.00	0.00	0.00	0.00
III	393	0.03	0.09	0.00	0.50
**IV**	**393**	**0.94**	**0.12**	**0.50**	**1.00**
V	393	0.03	0.08	0.00	0.49
I	185	0.00	0.00	0.00	0.00
II	185	0.00	0.00	0.00	0.00
III	185	0.00	0.00	0.00	0.00
IV	185	0.03	0.08	0.00	0.46
**V**	**185**	**0.97**	**0.08**	**0.54**	**1.00**

Legend: n=group size. Mean, SD, Min and Max, are the mean, stadndrad deviation, minimum and maximum group membership probabilities.

Table S2 (Supplementary)

Unadjusted and Adjusted§ estimates of diffrences of baseline clinical indicators between Group I and other trajectories (Group II, III, IV and V)

Risk [illegible]	Unadjusted estimates			Adjusted§			
	Coefficient	[95% CI]		Coefficient	[95% CI]		**P value**
eGFR							
I	Reference			Reference			
II	2.86	0.18	5.53	3.06	0.45	5.67	**0.02**
III	16.49	13.89	19.08	14.54	11.97	17.12	**0.00**
IV	32.63	30.00	35.26	28.26	25.52	31.00	**0.00**
V	52.44	49.47	55.41	45.17	41.90	48.43	**0.00**
HbA1C							
I							
II	-9.29	-6.43	-0.07	-8.49	-12.80	-4.17	**0.000**
III	-8.42	-8.98	-2.82	-8.68	-12.93	-4.43	**0.000**
IV	-8.65	-	-6.89	-11.46	-15.98	-6.94	**0.000**
V	-4.74	-	-9.54	-10.53	-15.92	-5.14	**0.000**
Systolic BP							
I							
II	-3.25	-6.43	-0.07	-3.69	-6.81	-0.57	**0.020**
III	-5.90	-8.98	-2.82	-4.66	-7.74	-1.58	**0.003**
IV	-10.02	-	-6.89	-6.46	-9.73	-3.19	**0.000**
V	-13.07	-	-9.54	-6.79	-10.69	-2.89	**0.001**
Diastolic BP							
I							
II	-2.02	-3.86	-0.18	-2.29	-4.12	-0.46	**0.014**
III	-1.76	-3.54	0.01	-1.83	-3.64	-0.03	**0.047**
IV	-2.06	-3.87	-0.26	-1.69	-3.61	0.23	**0.084**
V	-2.70	-4.74	-0.66	-1.98	-4.27	0.31	**0.090**
ACR							
I							
II	-16.72	-	-9.13	-15.94	-23.42	-8.46	**0.000**
III	-25.43	-	-18.08	-27.47	-34.85	-	**0.000**
IV	-27.03	-	-19.57	-33.86	-41.70	-	**0.000**
V	-31.28	-	-22.86	-44.01	-53.35	-	**0.000**
BMI							
I							
II	-0.22	-1.16	0.71	-0.19	-1.13	0.74	**0.687**
III	-0.77	-1.67	0.14	-0.57	-1.49	0.36	**0.229**
IV	-0.93	-1.85	-0.01	-0.57	-1.55	0.41	**0.250**
V	-1.71	-2.75	-0.67	-1.14	-2.30	0.03	**0.057**
Triglycerides							
I							
II	-0.07	-0.27	0.13	-0.11	-0.31	0.08	**0.258**
III	-0.15	-0.35	0.04	-0.21	-0.41	-0.02	**0.031**
IV	-0.19	-0.38	0.01	-0.24	-0.44	-0.03	**0.024**
V	-0.24	-0.46	-0.02	-0.31	-0.56	-0.07	0.013

Table S2 (Supplementary) continue..

Risk	Coefficient	[95% CI]		Coefficient	[95% CI]		**P value**
HDL							
I	Reference			Reference			
II	-0.04	-0.14	0.06	0.00	-0.09	0.10	**0.945**
III	-0.05	-0.14	0.05	-0.04	-0.13	0.06	**0.422**
IV	0.01	-0.09	0.11	-0.04	-0.14	0.06	**0.466**
V	0.07	-0.04	0.18	-0.03	-0.15	0.09	**0.636**
LDL							
I							
II	-0.08	-0.26	0.09	-0.07	-0.25	0.11	**0.441**
III	0.01	-0.16	0.18	-0.00	-0.18	0.17	**0.985**
IV	0.02	-0.15	0.20	-0.01	-0.20	0.17	**0.896**
V	-0.12	-0.31	0.08	-0.20	-0.42	0.02	**0.081**
Cholesterol							
I							
II	-0.07	-0.28	0.15	-0.04	-0.26	0.17	**0.685**
III	-0.05	-0.26	0.16	-0.07	-0.29	0.14	**0.489**
IV	-0.11	-0.32	0.10	-0.19	-0.41	0.04	**0.101**
V	-0.23	-0.47	0.00	-0.38	-0.65	-0.11	**0.005**

Table S3(Supplementary)

Summary of missing data (out of 11 time points) over time by trajectory

TIME	I	II	III	IV	V	total
0 (Baseline)	60	115	106	106	47	434
	47.24	33.43	23.77	26.97	25.41	29.03
1	26	72	122	108	55	383
	20.47	20.93	27.35	27.48	29.73	25.62
2	14	60	71	78	29	252
	11.02	17.44	15.92	19.85	15.68	16.86
3	9	43	46	28	22	148
	7.09	12.50	10.31	7.12	11.89	9.90
4	9	22	44	36	15	126
	7.09	6.40	9.87	9.16	8.11	8.43
5	6	12	26	15	10	69
	4.72	3.49	5.83	3.82	5.41	4.62
6	2	9	19	13	1	44
	1.57	2.62	4.26	3.31	0.54	2.94
7	1	7	9	5	4	26
	0.79	2.03	2.02	1.27	2.16	1.74
8	0	3	2	4	0	9
	0.00	0.87	0.45	1.02	0.00	0.60
9	0	1	1	0	2	4
	0.00	0.29	0.22	0.00	1.08	0.27
Total	127	344	446	393	185	1495

Table S4 (Supplementary)

Kappa and weighted Kappa to assess the agreement of the classification achieved by the final model and other models using complete data and less number of missing

Missing Data (baseline to year 10)	Method	Agreement	Expected agreement	Kappa	Se	Z value	P-value
0	Kappa	91.94%	22.03%	0.90	0.03	35.82	0.000
	Wt. Kappa	97.98%	65.82%	0.94	0.03	29.65	0.000
≤ 1	Kappa	94.98%	22.52%	0.94	0.02	50.74	0.000
	Wt. Kappa	98.75%	66.81%	0.96	0.02	42.06	0.000
≤ 2	Kappa	96.73%	22.89%	0.96	0.02	58.89	0.000
	Wt. Kappa	96.73%	22.89%	0.96	0.02	58.89	0.000
≤ 3	Kappa	96.88%	23.01%	0.96	0.02	62.73	0.000
	Wt. Kappa	99.22%	67.53%	0.98	0.02	51.92	0.000
≤ 4	Kappa	98.81%	23.12%	0.98	0.01	67.49	0.000
	Wt. Kappa	99.70%	67.71%	0.99	0.02	55.48	0.000
≤ 5	Kappa	99.43%	23.0%	0.99	0.01	69.83	0.00
	Wt. Kappa	99.86%	68.0%	1.00	0.02	57.27	0.00

Table S5 (Supplementary) Sumarry of individiual slopes (excluding observations that follow eGFR ≤ 15 ml/min/1.73m^2) for each of the five groups

Group	Data	Group size	Mean slope	SD	Min	Max
I	**1**	126	-3.52	3.64	-17.50	1.29
	**2**	126	-0.09	0.11	-0.61	0.03
II	**1**	343	-0.07	1.65	-7.71	7.50
	**2**	343	0.00	0.03	-0.14	0.13
III	**1**	445	0.54	1.72	-17.04	4.43
	**2**	445	0.01	0.02	-0.32	0.06
IV	**1**	393	0.51	1.39	-4.29	5.14
	**2**	393	0.01	0.01	-0.05	0.05
V	**1**	183	-0.20	1.48	-6.98	3.39
	**2**	183	0.01	0.01	-0.09	0.03

Note. SD:standard deviation. 1:original data, 2:log transformed data. Slopes were calculated for groups with at least 3 observation per patinet. 10 Particpants were dropped as they did not meet the criterion.

Table S6 (Supplementary)

Proportions of people with Type I diabetes reching various CKD stages over 10 years of follow up by trajectory

Group	Reference group	Comparison group	eGFR estimates				CKD stage
(1)	eGFR ≥ 45 (baseline)	eGFR < 60 (year 10)	Mean	SD	Min	Max	(CKD-Stage 3A)%
I	127	125	34.23	14.69	4.60	57.8	98.43%
II	344	125	50.27	7.66	23.80	59.9	36.34%
III	446	11	54.98	3.67	47.80	59.42	2.47%
IV	393	0					0.00%
V	185	1	35.40	.	35.40	35.4	0.54%
(2)	eGFR ≥ 60 (baseline)	eGFR < 60 (year 10)					(CKD-Stage 3A)%
I	56	55	32.08	15.07	4.60	57.2	98.21%
II	225	72	49.25	8.18	24.40	59.9	32.00%
III	425	11	54.98	3.67	47.80	59.42	2.59%
IV	389	0					0.00%
V	185	1	35.40	.	35.40	35.4	0.54%
(3)	eGFR ≥ 45 (baseline)	eGFR < 45 (year 10)					(CKD Stage 3B)%
I	127	91	28.13	12.64	4.60	44.88	71.65%
II	344	20	36.66	6.70	23.80	44.9	5.81%
III	446	0					0.00%
IV	393	0					0.00%
V	185	1	35.40	.	35.40	35.4	0.54%
(4)	eGFR ≥ 45 (baseline)	eGFR < 30 (year 10)					(CKD stage 4) %
I	127	43	16.77	8.04	4.6	29.80	33.86%
II	344	4	26.24	2.57	23.8	29.17	1.16%
	eGFR ≥ 45 (baseline)	eGFR < 15 (year 10)					(CKD stage 5) %
I	127	21	9.79	3.38	4.6	14.90	16.54%

Figure S1 (Supplementary)

Historgrams of individual slopes of eGFR over 10 years by trajectories (groups)

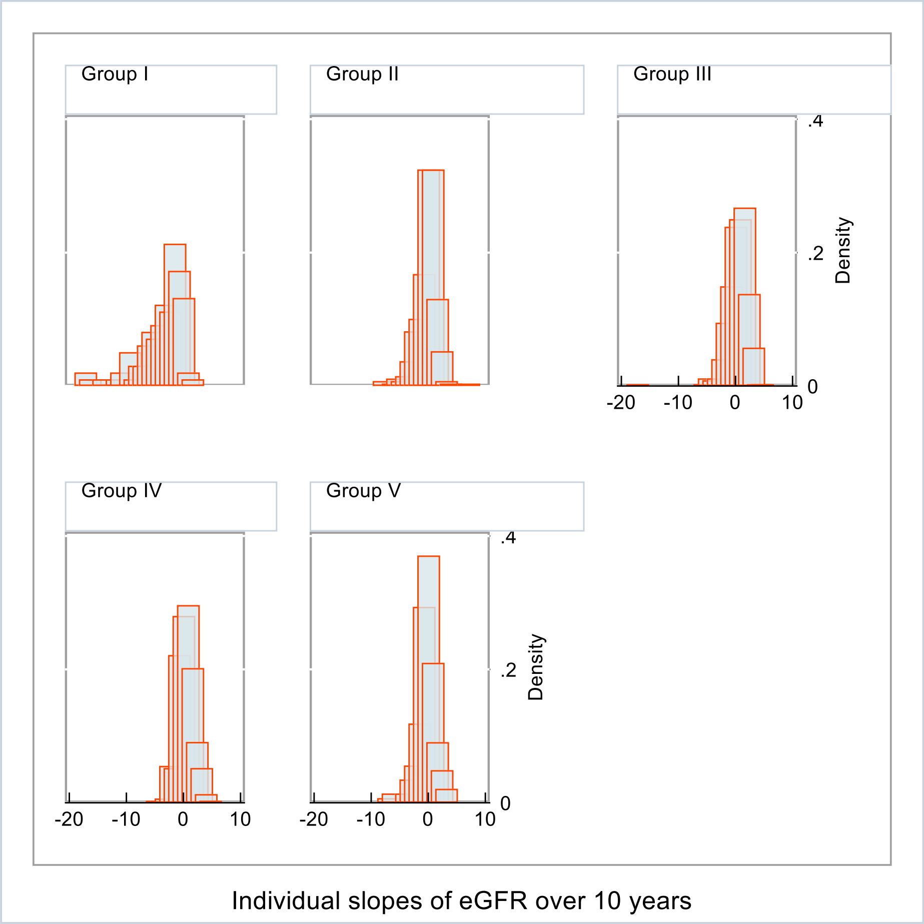

Note. Values following eGFR ≤15 were treated as missing
