# Supplementary material for: Ten years trajectories of estimated glomerular filtration rate (eGFR) in a multiethnic cohort of people with type 1 diabetes and preserved renal function
Source: BMJ Open. 2024 Sep 10;14(9):e083186. doi: 10.1136/bmjopen-2023-083186 (PMC11409247; doi:10.1136/bmjopen-2023-083186)
Supplement: Supplementary file 2 [file bmjopen-2023-083186.reviewer_comments.pdf]

PEER REVIEW HISTORY

BMJ Open publishes all reviews undertaken for accepted manuscripts. Reviewers are asked to complete a checklist review form and are provided with free text boxes to elaborate on their assessment. These free text comments are reproduced below.

ARTICLE DETAILS

**TITLE (PROVISIONAL)**	Ten years trajectories of estimated glomerular filtration rate (eGFR) in a multi-ethnic cohort of people with Type 1 diabetes and preserved renal function
**AUTHORS**	Ayis, Salma; Mangelis, Anastasios; Fountoulakis, Nikolaos; Collins, Julian; Alobaid, Thamer S; Gnudi, Luigi; Hopkins, David; Vas, Prashanth; Thomas, Stephen; Goubar, Aicha; Karalliedde, Janaka

VERSION 1 - REVIEW

**REVIEWER NAME**	***Berhe, Derbew Fikadu***
**REVIEWER AFFILIATION**	Addis Ababa University College of Health Sciences, School of Pharmacy, Dept of pharmacology and clinical pharmacy
**REVIEWER CONFLICT OF INTEREST**	Na
**DATE REVIEW RETURNED**	11-Jan-2024

**GENERAL COMMENTS**	The interaction between ethnicity, diabetes, and kidney function may present a multifaceted challenge in healthcare. T1DM, characterized by early-onset insulin deficiency, presents unique complexities in managing long-term complications like CKD. This paper investigates the potential role of ethnicity in influencing kidney function decline in T1DM patients. By examining the paper's findings, I herewith provide my input to the ongoing on T1DM care and its intersection with diverse ethnic backgrounds described on this paper.<br>Comment<br>I read the paper with interest and have a few comments.<br>1. In the abstract part, It would be good to write the primary and secondary outcomes separately.<br>2. In the method part, I wonder how authors classify patient's ethnicity. This is a very sensitive and difficult issue. In the authors' other paper (Ref #8), it stated that the ''local general population of southeast London is ethnically diverse, with >30% of people being of African Caribbean heritage.'' Authors need to make its clear how they define ethnicity. For example, what if a type 1 DM patient has white father/black mother (the other way round). What if some of patients have very heterogenous ethnicity?<br>3. In LMICs, prevalence of type I DM among children is increasing at an alarming rate. While most healthcare services focus on managing hyperglycaemia, there is limited effort to monitor kidney function. My first impression on the title of this paper was to see detail ethnic disparities in Type 1 DM-related kidney function decline among youngsters. However, average age of this study participants is almost above 30 years. Does that mean they exclude younger patients in the analysis? Or, Knowing the fact that Type I DM is juvenile on set, does that mean younger Type I DM patients in this study (<30 years old) do not have kidney function problems?<br>4. Overall, it is good to add some more details to the method section (though indicated at reference #8, difficult to comment on that paper).

**REVIEWER NAME**	***Azadnajafabad, Sina***
**REVIEWER AFFILIATION**	University of Leeds Faculty of Medicine and Health, Leeds Institute of Rheumatic and Musculoskeletal Medicine
**REVIEWER CONFLICT OF INTEREST**	Na
**DATE REVIEW RETURNED**	13-May-2024

GENERAL COMMENTS	Thanks for the chance to review this study. This study aimed to assess the estimated glomerular filtration rate (eGFR) patterns among a multi-ethnic cohort of individuals with Type 1 diabetes mellitus. The findings suggest distinct eGFR trajectories in TIDM individuals, with a higher risk observed among African Caribbean, highlighting the important role of ethnicity in disease progression. The study notion is interesting and via a robust methodology the obtained results are reliable to inform the clinicians. However, some comments need to be addressed to make everything clear and sound. Below, please find my comments and suggestions in this regard.<br>1. Abstract: please enrich the “setting” part about the cohort characteristics and follow-up intervals as it is important in interpretation of the findings provided in results in abstract.<br>2. Background: before introducing the topic of eGFR progression in T1DM patients, it is essential to talk about the epidemiology and pathophysiology of CKD in these patients and why we need to study this important complication of diabetes. As most of the patients are younger populations, appropriate management of this fatal complication is necessary to improve the survival rates of affected patients.<br>3. The study cited as reference number 8 as the previous related should be expanded in the introduction and briefly the results be provided. Also, the differences between two studies need to be more clearly stated as both these studies rise from the same project and they share many parts of the methodology, but have different objectives.<br>4. Methods: the full list of clinical measures should be provided. We see authors mentioned the lack of medication history and habitual history as a limitation of their study. Was this true about the past medical history, too? Could the authors please explain how such a cohort did not capture such valuable variables in their records? This might affect the produced results and introduce unresolved confounders in the statistical analysis.<br>5. Statistical analysis: we understand that authors have used the linear regression as the base method to extract slopes for modelling. Could authors please explain about the assumptions of the regression and if it was correctly recruited to answer the questions of this study based on the results?<br>6. Statistical analysis: did authors consider any methods to validate the developed trajectory model like examining it on trained and tested datasets?<br>7. Table 1: the choice of presenting the continuous variables could be made based on their distribution via mean ± SD or median (IQR) and either one of them could be presented here and in the text.<br>8. Many typo errors exist through the text which needs appropriate amendments in future revisions.<br>9. Discussion: the observed results on the remarkable differences based on ethnicity needs more through discussion and justifications based on available literature.

VERSION 1 – AUTHOR RESPONSE

Reviewer: 1

Dr. Derbew Fikadu Berhe, Addis Ababa University College of Health Sciences

Comments to the Author:

Title: Exploring distinct groups of estimated glomerular filtration rate trajectories and their progression in a multi-ethnic cohort of people with Type 1 diabetes

The interaction between ethnicity, diabetes, and kidney function may present a multifaceted challenge in healthcare. T1DM, characterized by early-onset insulin deficiency, presents unique complexities in managing long-term complications like CKD. This paper investigates the potential role of ethnicity in influencing kidney function decline in T1DM patients. By examining the paper's findings, I herewith provide my input to the ongoing on T1DM care and its intersection with diverse ethnic backgroundsdescribed on this paper.

Comment

I read the paper with interest and have a few comments.

1. In the abstract part, It would be good to write the primary and secondary outcomes separately.

Thank you! As the method used does not differentiate between variables used in the construction of groups, and others used for between groups comparisons as distinctive primary and secondary but equally involved in the analysis and results, we have listed all endpoints in abstract and provided details in the manuscript method section.

2. In the method part, I wonder how authors classify patient's ethnicity. This is a very sensitive and difficult issue. In the authors' other paper (Ref #8), it stated that the ''local general population of southeast London is ethnically diverse, with >30% of people being of African Caribbean heritage.'' Authors need to make its clear how they define ethnicity. For example, what if a type 1 DM patient has white father/black mother (the other way round). What if some of patients have very heterogenous ethnicity?

Ethnicity was self-reported and was said in abstract and methods section. We used the participants definition of their ethnicity. Those with parents from two different ethnic groups are reported as mixed.

We have added a text to clarify this in Table 1, as mixed and others were combined due to small numbers, and a note was added to the table legend.

3. In LMICs, prevalence of type I DM among children is increasing at an alarming rate. While most healthcare services focus on managing hyperglycaemia, there is limited effort to monitor kidney function. My first impression on the title of this paper was to see detail ethnic disparities in Type 1 DM-related kidney function decline among youngsters. However, average age of this study participants is almost above 30 years. Does that mean they exclude younger patients in the analysis? Or, Knowing the fact that Type I DM is juvenile on set, does that mean younger Type I DM patients in this study (<30 years old) do not have kidney function problems?

Yes, in fact the study is confined to adults. We have made that clear in the revised copy, as we only included people aged 18 years and above at baseline.

4. Overall, it is good to add some more details to the method section (though indicated at reference #8, difficult to comment on that paper).

We have added more details about the previous study, and highlighted differences between the current and the previous one, cited as “8” for clarity, in response to this comment and a similar comment form reviewer 2. See paper, methods section. While reference “8” was cited, our aim was to allow interested readers to find out more about another relevant study had they wished to. We do not expect comments on that paper. Thank you.

Reviewer: 2

Dr. Sina Azadnajafabad, University of Leeds Faculty of Medicine and Health

Comments to the Author:

Thanks for the chance to review this study. This study aimed to assess the estimated glomerular filtration rate (eGFR) patterns among a multi-ethnic cohort of individuals with Type 1 diabetes mellitus. The findings suggest distinct eGFR trajectories in TIDM individuals, with a higher risk observed among African Caribbean, highlighting the important role of ethnicity in disease progression. The study notion is interesting and via a robust methodology the obtained results are reliable to inform the clinicians. However, some comments need to be addressed to make everything clear and sound. Below, please find my comments and suggestions in this regard.

1. Abstract: please enrich the “setting” part about the cohort characteristics and follow-up intervals as it is important in interpretation of the findings provided in results in abstract.

Thank you, the duration of the study has been made clearer as are the timelines. This is now updated in the revised setting section of the abstract.

2. Background: before introducing the topic of eGFR progression in T1DM patients, it is essential to talk about the epidemiology and pathophysiology of CKD in these patients and why we need to study this important complication of diabetes. As most of the patients are younger populations, appropriate management of this fatal complication is necessary to improve the survival rates of affected patients.

See paper, Background has been edited and changes made according to reviewer’s suggestion, and 3 references added.

3. The study cited as reference number 8 as the previous related should be expanded in the introduction and briefly the results be provided. Also, the differences between two studies need to be more clearly stated as both these studies rise from the same project and they share many parts of the methodology, but have different objectives.

Thank you.

We have now reported in brief our previous study results and made clearer the differences and distinctions between previous study and the hypothesis and data analyses we reported in this manuscript.

Please see paper, methods section.

4. Methods: the full list of clinical measures should be provided.

That was provided in the methods section. We added clarity explaining that standardised clinical processes were used.

We see authors mentioned the lack of medication history and habitual history as a limitation of their study. Was this true about the past medical history, too? Could the authors please explain how such a cohort did not capture such valuable variables in their records? This might affect the produced results and introduce unresolved confounders in the statistical analysis.

This is a retrospective study, details on medication from these two factors were not sufficient to be used in the analysis. However, the methods used [Group Based Trajectory Models (GBTMs)] to identify groups which is the primary objective, do not require more than one outcome which has been used, eGFR. Later comparisons of outcomes between different groups would indeed have benefited from such missing data. While we do not have specific reasons for this missingness, it is not uncommon to have limited data on smoking / tobacco use, as patients may choose not to report these, or to provide inaccurate estimates of smoking habits. The points were clearly addressed as limitations in the discussion section, also now available in the added section about Strengths and limitations.

5. Statistical analysis: we understand that authors have used the linear regression as the base method to extract slopes for modelling. Could authors please explain about the assumptions of the regression and if it was correctly recruited to answer the questions of this study based on the results?

We have added clarity to the procedure used, alongside assumptions made and methods used for testing these assumptions to ensure good fitness of the models used, and to justify optimal solutions. Please see revised statistical analysis section.

6. Statistical analysis: did authors consider any methods to validate the developed trajectory model like examining it on trained and tested datasets?

Sensitivity analysis was used to test the robustness of the models based on number of missingness in the eGFR. This would serve as internal validation. External validation was recommended for further research with a similar multiethnicity cohort, as per the conclusion. We clarified this further in methods and results section.

7. Table 1: the choice of presenting the continuous variables could be made based on their distribution via mean ± SD or median (IQR) and either one of them could be presented here and in the text.

Table has been edited.

8. Many typo errors exist through the text which needs appropriate amendments in future revisions.

The article has been proofread and carefully revised for grammar and typos errors. We colour marked these where possible in the marked copy. Thank you!

9. Discussion: the observed results on the remarkable differences based on ethnicity needs more through discussion and justifications based on available literature.

Discussion section has been revised to cover issues raised by this point. Please See paper

Reviewer: 1

Competing interests of Reviewer: NA

Reviewer: 2

Competing interests of Reviewer: I have no competing interests.

VERSION 2 – REVIEW

**REVIEWER NAME**	***Berhe, Derbew Fikadu***
**REVIEWER AFFILIATION**	Addis Ababa University College of Health Sciences, School of Pharmacy, Dept of pharmacology and clinical pharmacy
**REVIEWER CONFLICT OF INTEREST**	Na
**DATE REVIEW RETURNED**	27-Jul-2024

**GENERAL COMMENTS**	All my comments are well addressed.<br><br>It is good the self-reporting ethnicity as one potential limitation.

**REVIEWER NAME**	Azadnajafabad, Sina
**REVIEWER AFFILIATION**	University of Leeds Faculty of Medicine and Health, Leeds Institute of Rheumatic and Musculoskeletal Medicine
**REVIEWER CONFLICT OF INTEREST**	Na
**DATE REVIEW RETURNED**	09-Jul-2024

**GENERAL COMMENTS**	The authors successfully addressed the comments to an acceptable extent. I have no further comments or suggestions.
